# Ibrutinib combined with rituximab and high-dose methotrexate in newly diagnosed primary CNS diffuse large B-cell lymphoma: a pilot study with long-term follow-up

**DOI:** 10.3389/fonc.2025.1669385

**Published:** 2025-10-02

**Authors:** Yixian Guo, Yongzhi Shan, Yueshan Piao, Xiaoli Chang, Dongmei Zou, Qiang Ma, Yukui Wei, Geng Xu, Yaming Wang, Dandan Wang, Lianghong Teng, Chunxue Wu, Zhilian Zhao, Tianbin Song, Hong Zhao, Wuhan Hui, Li Su, Wanling Sun

**Affiliations:** ^1^ Department of Hematology, Xuanwu Hospital, Capital Medical University, Beijing, China; ^2^ Department of Neurosurgery, Xuanwu Hospital, Capital Medical University, China International Neuroscience Institute, Beijing, China; ^3^ Department of Pathology, Xuanwu Hospital, Capital Medical University, Beijing, China; ^4^ Department of Radiology, Xuanwu Hospital, Capital Medical University, Beijing, China

**Keywords:** newly diagnosed, primary diffuse large B-cell lymphoma of the CNS, Bruton tyrosine kinase inhibitor, methotrexate, ibrutinib

## Abstract

**Key point:**

IRM appears promising and well tolerated as first-line therapy for newly diagnosed PCNS DLBCL in a small pilot cohort; these hypothesis-generating results require confirmation in larger prospective studies.

**Background:**

Primary diffuse large B-cell lymphoma of the central nervous system (PCNS DLBCL) is a rare, aggressive lymphoma with rising incidence in elderly patients. Bruton tyrosine kinase (BTK) inhibitors show promise in recurrent/refractory cases, warranting exploration in newly diagnosed disease.

**Methods:**

This single-center pilot study evaluated the safety/efficacy of ibrutinib, rituximab, and high-dose methotrexate (IRM) in nine newly diagnosed PCNS DLBCL patients (2018–2019). Treatment included 4 cycles of IRM induction, consolidation (HSCT or 2 additional IRM cycles), and maintenance therapy (ibrutinib/lenalidomide).

**Results:**

After induction, overall response rate (ORR) was 100% (complete response [CR]: 77.8%, partial response [PR]: 22.2%). Post-consolidation, CR increased to 88.9%. At a median follow-up of 77.6 months, 5-year overall survival (OS) and progression-free survival (PFS) rates were both 77.8%, with 8 patients in sustained CR and one progression. No treatment-related deaths occurred; grade ≥3 adverse events were rare (2 neutropenia, 2 anemia, 1 gastrointestinal bleeding).

**Conclusion:**

In this small pilot cohort, IRM showed promising activity and tolerability as first-line therapy for PCNS DLBCL. These descriptive findings warrant confirmation in larger prospective trials (#ChiCTR1900027811).

## Introduction

Primary diffuse large B-cell lymphoma of the CNS (PCNS DLBCL), a rare variant of extranodal non-Hodgkin lymphoma (NHL), affects the brain, leptomeninges, eyes, or spinal cord without the involvement of systemic manifestations (1). Regardless of the low annual incidence of PCNS DLBCL (0.4/100,000), the cases are rising, especially among the elderly population (2). Although the overall median survival time has doubled from 12.5 months to 26 months (1970s to 2010s), nearly 50% of patients experience disease relapse within 2 years. Patients under 70 years of age have some benefits but elderly people face persistent challenges (3–5). High-dose methotrexate (HD-MTX) remains the backbone of induction therapy, but consensus on optimal regimens is lacking (6–9). Consolidation therapy, including autologous stem cell transplantation (ASCT) and whole-brain radiotherapy (WBRT), is not quite used in elderly PCNS DLBCL patients (10, 11).

The NF-κB pathway, constitutively activated in PCNS DLBCL through mutations in MYD88 and CD79B, drives tumor survival via Bruton’s tyrosine kinase (BTK)-mediated B-cell receptor (BCR) signaling (12, 13). Ibrutinib, a covalent BTK inhibitor, demonstrates potent activity in relapsed/refractory PCNS DLBCL, achieving cerebrospinal fluid (CSF) concentrations exceeding the BTK IC (IC, inhibitory concentration) (14–16). Preclinical studies further suggest synergy between ibrutinib and HD-MTX via inhibition of the breast cancer resistance protein (BCRP), enhancing systemic MTX exposure (17). Furthermore, previous research combining ibrutinib with chemotherapy, excluding HD-MTX, reported significant treatment-related toxicity, such as pulmonary and cerebral aspergillosis (18). These findings provide a mechanistic rationale for combining ibrutinib with HD-MTX and rituximab (IRM) in induction therapy. Our study also includes consolidation therapy post-induction, involving ASCT or two additional cycles of IRM. Furthermore, maintenance therapy with oral ibrutinib or lenalidomide is administered subsequently.

In this pilot study, we evaluated the long-term safety and efficacy of IRM in newly diagnosed PCNS DLBCL. With a median follow-up of 77.6 months, our results demonstrate durable responses and favorable survival outcomes.

## Methods

### Study design

This single-center, retrospective pilot study aimed to evaluate the feasibility, safety, and preliminary efficacy of the IRM regimen (ibrutinib, rituximab, and HD-MTX) in newly diagnosed PCNS DLBCL. The study was conducted at Xuanwu Hospital, Capital Medical University, between April 2018 and May 2019. As a pilot study, the primary focus was on generating hypotheses and assessing the tolerability of the IRM regimen, with the goal of informing future larger-scale, prospective trials.

### Ethics statement

This study was conducted in accordance with the Declaration of Helsinki and was approved by the Ethics Committee of Xuanwu Hospital, Capital Medical University (Approval No. [2019]079). The ethics committee approved both the entire IRM treatment protocol and the retrospective analysis of anonymized patient data. Informed consent was obtained from all participants for the use of their anonymized clinical data for research purposes.

### Participants

A total of 9 patients with newly diagnosed PCNS DLBCL were enrolled for the IRM regimen in the Department of Hematology, Xuanwu Hospital, Capital Medical University, between April 2018 and May 2019. The diagnosis of PCNS DLBCL was performed according to the WHO 2016 diagnostic criteria for hematopoietic and lymphoid tissue tumors (19). All patients were diagnosed by histopathological examination of biopsied brain lesions and underwent a complete extent-of-disease evaluation, including PET-CT scans, bone marrow morphology, bone marrow flow cytometry, and bone marrow IgH rearrangement analysis, to exclude systemic lymphoma or document involvement of the eyes or CSF. Cerebrospinal fluid (CSF) analysis, including cytology and flow cytometry for lymphoma cells, was performed in 7 patients at diagnosis; no monoclonal B lymphocytes were detected in any of the patients tested.; all had parenchymal brain lesions. CSF analysis was performed in 7 of the 9 patients; the remaining 2 patients did not undergo CSF analysis due to disease-related factors. Inclusion criteria included previously untreated PCNS DLBCL with at least one measurable lesion and the age of ≥18 and ≤70 years. Eligible patients had adequate organ function and LVEF of ≥50% measured by cardiac echocardiography. Patients with gastrointestinal perforation and/or fistula within 6 months prior to enrollment or concurrent serious non-malignant disease impacting the compliance with the study protocol, such as serious cardiovascular disease, uncontrolled diabetes and hypertension, and HIV antibody positive, HCV antibody positive, HBV surface antigen positive and HBV-DNA of >10^3^ copies were excluded.

### Clinical information

Patient characteristics such as gender, age, Eastern Cooperative Oncology Group (ECOG) score, prognostic indices (IELSG and MSKCC), lactate dehydrogenase (LDH) level, pathological type, treatment regimen, adverse reactions, and survival time, were collected. Clinical follow-up, imaging assessments, and survival calculations were performed until December 31, 2024.

### Treatment protocol

The IRM regimen consisted of the following components: Rituximab: 375 mg/m² administered intravenously on day 0;HD-MTX: 3.5 g/m² infused intravenously over 3 hours on day 1;Ibrutinib: A daily oral dose of 560 mg initiated once the plasma MTX concentration fell below 0.1 µmol/L, prior to the next HD-MTX dose. Each treatment cycle spanned 4 weeks (28 days). The 4-week interval was chosen to allow for adequate recovery from potential toxicities, particularly myelosuppression, and to minimize the risk of cumulative toxicity associated with repeated high-dose MTX administration. This interval was based on clinical experience and patient tolerance, while maintaining therapeutic efficacy. Following 4 cycles of IRM induction therapy, patients who achieved complete response (CR) or partial response (PR) proceeded to consolidation therapy. Consolidation options included: High-dose chemotherapy (HDC) followed by ASCT, or two additional cycles of the IRM regimen. Following consolidation therapy, patients received maintenance treatment with either ibrutinib (560 mg orally once daily) or lenalidomide (25 mg orally on days 1–21 of a 28-day cycle). The choice between these agents was based on patient preference, with lenalidomide supported by its demonstrated CSF penetration and efficacy in prolonging PFS among PCNSL patients, particularly in elderly populations (20, 21) Maintenance therapy continued until disease progression, intolerance, or for a maximum duration of 2 years. Ibrutinib and lenalidomide are not FDA-approved for PCNS DLBCL, use was approved by the Xuanwu Hospital Ethics Committee (No. [2019]079).

### DNA isolation and next-generation sequencing

Twenty formalin-fixed paraffin-embedded (FFPE) tissue samples were obtained from archived materials. DNA was extracted from the tissue samples according to the manufacturer’s instructions (BioTeke Corporation, Beijing, China), and stored at -20°C. Sufficient amounts of DNA for further analysis were obtained from all archived FFPE samples. Mutations on 47 genes involved in B cell lymphoma were analyzed by gene sequencing. The genes analyzed were *ARIDIA*, *B2M*, *BCL2*, *BIRC3*, *BRAF*, *CARD11*, *CCND1*, *CCND3*, *CD58*, *CD79A*, *CD79B*, *CLITA*, *CREBBP*, *CXCR4*, *DTX*, *EBF1*, *CP300*, *EZH2*, *FOX01*, *GNA13*, *MYD88*, *NTOCH2*, *PIMA*, *PTEN*, *SF3B1*, *SPEN*, *TCF3*, *TNFAIp3*, *XPO1*, *ATM*, *JAK3*, *NOTCH1*, *PTPN1*, *SOCS1*, *STST6*, *TET2*, and *TP53*. This method detected the hotspot regional variations associated with hematological lymphoma reported in COSMIC, including point mutations and short-fragment insertions or deletions, and could not detect gene copy number variations and fusion genes; the lower limit of detection for hotspot variations was 1% and for non-hotspot variations was 3%.The target fragment was amplified, purified, and connected to an adapter, followed by library enrichment and quality control according to the manufacturer’s instructions (Shanghai Yuanqi Biomedical Technology Co., Ltd., Shanghai, China). DNA was sequenced using the Illumina Miseq sequencer (average sequencing depth 1000X, pair-end 150 bp). Mutations were analyzed using COSMIC, ClinVar, HGMD, ExAC, and Ensembl.

### Assessment of therapeutic efficacy

Patients were evaluated after completion of every two cycles of IRM therapy, including gadolinium-enhanced brain magnetic resonance imaging (MRI) scans. The whole-body PET-CT scan was performed after four cycles of therapy. Assessment of therapeutic response was performed according to the International Primary CNS Lymphoma Collaborative Group (IPCG) 2005 criteria (22). Briefly, a CR was defined as complete disappearance of all enhanced lesions on MRI; a PR was defined as more than 50% reduction in the enhanced lesions; a PD was defined as more than 25% increase in the enhanced lesions or the reappearance of new lesion at the original site of the lesion, or at a new location after CR; and a stable disease (SD) included all the statuses other than CR, PR, and PD. Response assessment was performed at 4 months, at the end of consolidation therapy, and during maintenance.

Long-term outcomes were PFS and OS. PFS was defined as the duration from the study initiation date to the occurrence of any lymphoma relapse or death from any cause. Enhanced MRI imaging of the skull was evaluated once every 12 weeks after the end of the induction therapy until 2 years or the endpoint that was from the treatment of the patient until death.

### Side effects and complications

Therapy-related toxicity and complications were carefully evaluated and treated. Adverse events were documented and classified by the National Cancer Institute Common Terminology Standards for Adverse Events (NCI CTCAE version 4.03).

### Statistical analysis

OS was defined as the time interval from the date of the onset of the study to the death resulting from any cause or to the last follow-up. The Kaplan-Meier method was employed to estimate OS and PFS. Time was censored at the last follow-up date if no failure was observed. Patients who remained alive and in remission were censored at the time of their last follow-up. Data were analyzed using MedCalc software. Descriptive characteristics were summarized as qualitative indicators.

## Results

### Patient characteristics

In this pilot study, a total of 9 patients, comprising 7 men and 2 women, were included. The median age at the time of diagnosis was 59 years (48-66), and the median Karnofsky Performance Status (KPS) score was 40 (20-40).The low KPS scores observed in some patients (as low as 20) were attributable to tumor-related neurological deficits rather than systemic comorbidities; all patients had adequate major organ function and were thus eligible for intensive chemotherapy. Deep brain structure involvement was observed in six patients (66.9%). Pathological diagnosis confirmed that all patients had DLBCL, with 8 classified as non-GCB subtypes and 1 as GCB subtype. The median follow-up time was 77.6 (10.6 to 80.7) months (**Table 1**).

**Table 1:** Characteristics of the 9 patients in the I-RM pilot study.

Characterstics	Case series(N=9)
Ages-yr	
Median	57
Range	48-66
Sex-n.	
Male	7
Female	2
ECOG (Score)	
Median	3
Range	3-4
KPS (Score)	
Median	40
Range	20-40
Increased LDH - n.	0
High CSF protein concentrations - n*	6/7
Multiple lesions - n.	3
Deep lesions - n.	6
Leptomeningeal/Parenchymal involvement - n	0/9
Hans algorithm - n.	
GCBDLBCL	1
non-GCBDLBCL	8
Median Ki-67	
Median	80
Range	60-90
PETSUVmax	
Median	23.05
Range	12.7-45.57
IELSG-n.	
Low risk	0
Intermediate risk	6/7
High risk	1/7
MSKCC-n.	
Low risk	1
Intermediate risk	0
High risk	8
Median follow-up time (M)	77.6(10.6to80.7)

ECOG, Eastern Cooperative Oncology Group Performance Status; KPS, Karnofsky Performance Score; IELSG, International Extranodal Lymphoma Study Group; MSKCC score, Memorial Sloan Kettering Cancer Center model.

*Out of the 9 patients, 7 underwent lumbar puncture examinations and CSF analysis.

### Survival analysis

All patients completed 4 cycles of IRM induction therapy. The response to the induction therapy in 9 patients was as follows. The ORR was 100%, with 7 patients who achieved CR (77.8%) and 2 PR (22.2%). Among the 7 cases with CR, three exhibited minimal enhancement at the margin of the resection cavity on enhanced MRI scans, but no hypermetabolic lesions were observed on PET-CT scans. During subsequent follow-ups, there was no significant change in the minimal enhancement at the margin of the resection cavity on enhanced MRI scans. CRu was confirmed by two radiologists who specialized in imaging assessment. Consolidation therapy was ASCT in 3 patients and 2 cycles of IRM in 6 patients. The hematopoietic stem cell mobilization regimen for two patients involved high-dose cyclophosphamide and etoposide mobilization, while one patient opted for rituximab, high-dose methotrexate, and high-dose cytarabine. All three patients received the BEAC (carmustine, etoposide, cytarabine, cyclophosphamide). regimen as the conditioning regimen for ASCT. Notably, thiotepa was not commercially available in China during the years 2018-2019. Consequently, a thiotepa-containing transplant conditioning regimen was utilized for the subsequent prospective clinical trial in our center. However, six patients were unable to receive ASCT, primarily due to factors such as advanced age or refusal of transplantation.

**Figure 1:**
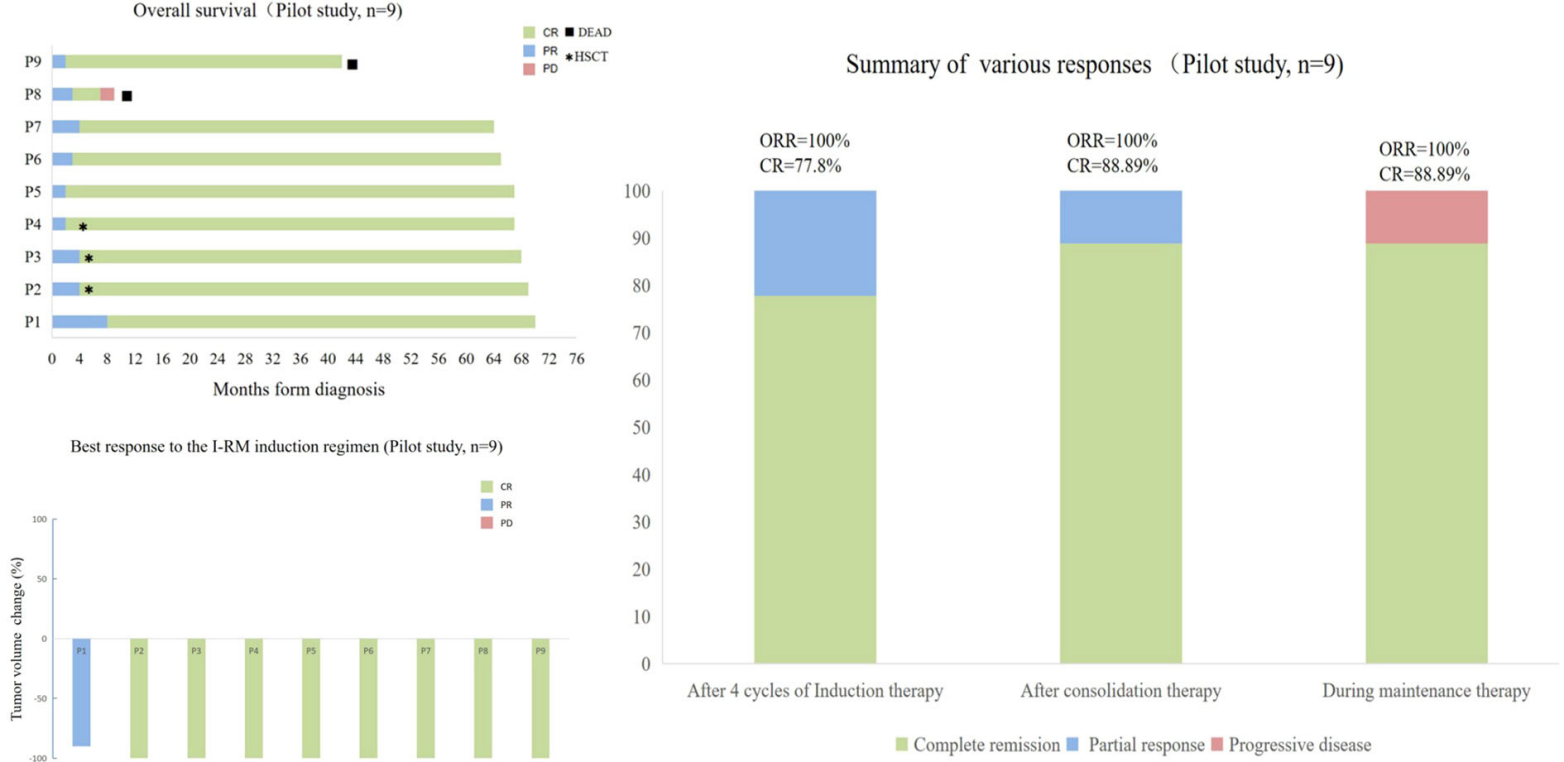
Treatment Responses of 9 Newly Diagnosed PCNS DLBCL Patients in the Study. **(A)** Overall survival of the 9 patients. **(B)** Best response to IRM induction regimen of the 9 patients. **(C)** Efficacy assessment of the 9 patients at the end of each stage of induction therapy, consolidation therapy, and maintenance therapy.

**Figure 2:**
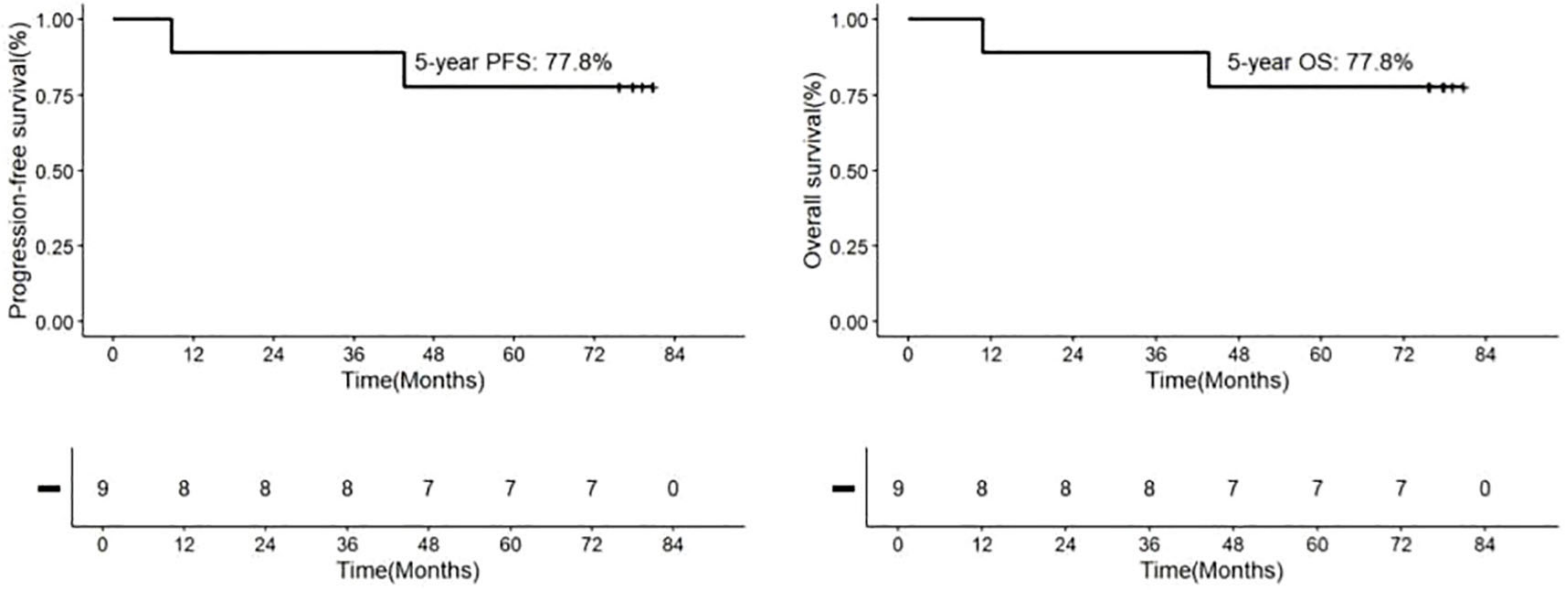
Kaplan-Meier Analyses of Outcomes in Patients with Newly Diagnosed PCNS DLBCL Receiving IRM Treatment. The 5-year rates are provided as means±standard error. Progression-Free Survival (PFS): 77.8%.

Following consolidation therapy, 8 out of 9 evaluable patients achieved CR, accounting for 88.9% of the cohort. Among the 9 patients, 8 chose maintenance therapy, while 1 patient did not receive lenalidomide or ibrutinib for maintenance due to proteinuria and a kidney biopsy suggesting autologous HSCT-related thrombotic microangiopathy (TMA) (23). During the maintenance therapy phase, one patient achieved CR from PR, while another patient had a sudden progressive and subsequently passed away 10.8 months after the initial diagnosis. Additionally, one patient died from a non-progressive-related grand mal seizure after the discontinuation of lenalidomide maintenance therapy and one year of oral sodium valproate, 43.6 months after the initial diagnosis. One patient discontinued the maintenance therapy due to other medical conditions. In summary, among the 9 newly diagnosed PCNS DLBCL patients, the final response included 8 cases of CR (88.9%) and 1 case of PD (11.1%) (**Figure 1** and **Table 2**). Median PFS and OS were not reached and the 5-year OS and PFS were 77.8% and 77.8% respectively (**Figure 2**).

**Table 2:** Treatment-related adverse events by treatment phase.

Adverse events	Induction phase n=9 (No. [%])			Consolidation phase IRM n=6 (No. [%])	Consolidation phase ASCT† n=3 (No. [%])	Maintenance phase (Ibrutinib) n=2 (No. [%])	Maintenance phase (lenalidomide) n=6 (No. [%])	Treatment cessations
	Grade 1-2	Grade 3	Grade 4	Grade 3 and above	Grade 3 and above	Grade 3 and above	Grade 3 and above	
Hematologic toxicity								
Neutropenia	2(22.2%)	1(11.1%)	0	0	3(100%)	0	0	0
Anemia	2(22.2%)	2(22.2%)	0	0	3(100%)	0	0	0
Thrombocytopenia	1(11.1%)	0	0	0	3(100%)	0	0	0
Nonhematologic toxicity								
Hypertension	0	1(11.1%)	0	0	0	0	0	0
Atrial fibrillation	0	0	0	0	0	0	0	0
Mucositis oral	9(100%)	0	0	0	0	0	0	0
Vomiting and Nausea	9(100%)	0	0	0	3(100%)	0	0	0
Diarrhea	1(11.1%)	0	0	0	1(33.3%)	0	0	0
AST increased	2(22.2%)	0	0	0	0	0	0	0
ALT increased	3(33.3%)	0	0	0	0	0	0	0
Alk increased	0	0	0	0	0	0	0	0
Blood bilirubin increased	0	0	0	0	0	0	0	0
GGT increased	1(11.1%)	1(11.1%)	0	0	0	0	0	0
Creatinine increased	0	1	0	0	1(33.3%)	0	0	1
Pneumonitis	0	0	0	0	1(33.3%)	0	0	0
Gastrointestinal hemorrhage	1(11.1%)	1(11.1%)	0	0	0	0	0	0
hematuria	1(11.1%)	0	0	0	0	0	0	0

†Consolidation: All patients received consolidation therapy, consisting of 2 additional cycles of IRM (n = 6) or high-dose chemotherapy followed by ASCT (n = 3).

Toxicities for ASCT include those related to the conditioning regimen (BEAC) and the transplant procedure itself.

### Treatment compliance and toxicity

No treatment-related deaths occurred in patients who received IRM induction therapy and additional IRM consolidation cycles. The incidence of grade ≥3 treatment-related adverse events across different treatment phases is detailed in **Table 3**. Overall, the regimen was well-tolerated. During the induction phase, toxicity was primarily hematologic, including grade 3 neutropenia in 2 patients (22.2%) and grade 3 anemia in 2 patients (22.2%); non-hematologic toxicity included one case (11.1%) of grade 3 gastrointestinal hemorrhage associated with concomitant rivaroxaban use. Among the three patients undergoing consolidation with autologous stem cell transplantation (ASCT), all (100%) developed grade 3 neutropenia. Non-hematologic toxicities during the consolidation phase included pneumonitis, diarrhea, and elevated creatinine, each occurring in one patient (11.1%). In contrast, no grade ≥3 hematologic toxicities were observed in the six patients who received IRM consolidation. During the maintenance phase, toxicities were less frequent and manageable; one patient on lenalidomide developed grade 3 anemia, and one patient on ibrutinib required a dose reduction due to grade 3 limb pain. No cases of fungal infection or atrial fibrillation were observed in any phase. One patient did not receive maintenance therapy due to proteinuria and a kidney biopsy suggestive of transplant-associated thrombotic microangiopathy.

**Table 3:** Treatment response and mutation profile of nine PCNSL patients undergoing IRM therapy.

ID	Response to IRM introduction therapy	Consolidation therapy	Maintence therapy	Progressive disease	Survive	Hans	Mutations
#1	PR	IRM	Lenalidomide	N	live	non-GCB	MYD88/CARD11/CD79B/DTX1/PIM1/MYC/CREBBP
#2	CR	ASCT	Lenalidomide	N	live	non-GCB	MYD88/CD58/CD79B/DTX1/PIM1/SOCS1/CIITA
#3	CR	ASCT	None	N	live	non-GCB	CD79B/MEF2B/PIM1
#4	CR	ASCT	Lenalidomide	N	live	non-GCB	IRF8/GNA13/DTX1
#5	CR	IRM	Lenalidomide	N	live	non-GCB	MYD88/CD79B/PIM1
#6	CR	IRM	Ibrutinib	N	live	non-GCB	MYD88/BCL2/GNA13
#7	CR	IRM	Lenalidomide	N	live	non-GCB	ITPKB/CTITA/PIM1
#8	CR	IRM	Ibrutinib	Y	die	GCB	MYD88/CARD11/CD79B/B2M/EP300/TET2
#9	CR	IRM	Lenalidomide	N	die	non-GCB	PIM1/IRF8

CR, complete response; PR, partial response; ASCT, autologous stem cell transplantation.

### Next-generation sequencing analysis

**Figure 3:**
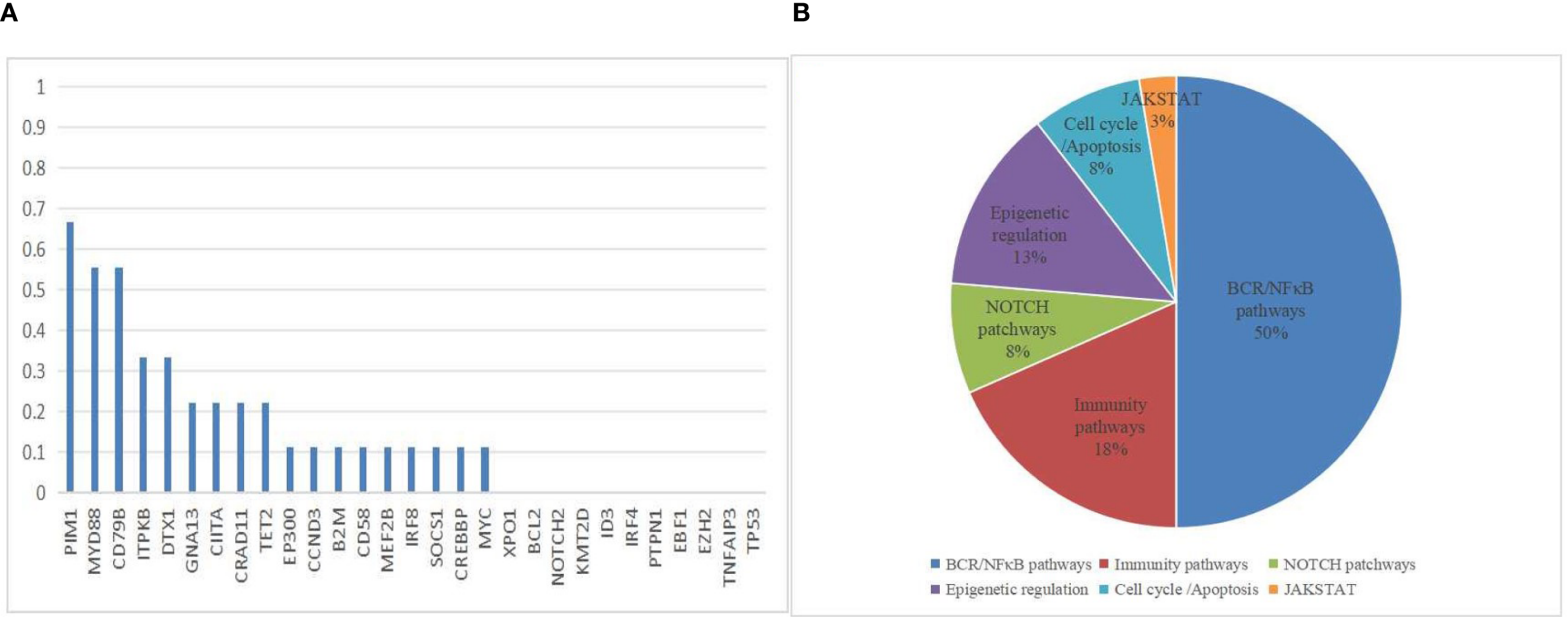
Mutation Frequencies in PCNSL Cohort. **(A)** The distribution and frequency of genetic alterations in 9 PCNSL specimens. **(B)** Mutation pathway distribution among PCNSL specimens. Different colors represent diverse mutation pathways.

NGS analysis of B-cell lymphoma gene mutations in 9 patients revealed a median of 3.5 (1-7) mutations per patient. The most common mutations were *PIM1* (66.7%), *MYD88 L265P* (55.6%), *CD79B* (55.6%), *ITPKB* (33.3%), *DTX1* (33.3%), *GNA13* (22.2%), *CIITA* (22.2%), *CARD11* (22.2%), and *TET2* (22.2%). Mutations in the genes related to the NF-κB signaling pathway accounted for 50% of cases, being the most frequent mutations in this pathway (**Figure 3**). due to limited data, no correlation analysis between treatment response and mutation data was performed. Due to limited patient number and data, no correlation analysis between treatment response and mutation data was performed. The genetic mutation profiles of the 9 patients are presented in **Table 2**.

## Discussion

In this pilot study, the IRM regimen (ibrutinib, rituximab, and HD-MTX) demonstrated promising outcomes in newly diagnosed primary diffuse large B-cell lymphoma of the central nervous system (PCNS DLBCL). The estimated 5-year OS and PFS rates were both 77.8%, with an ORR of 100% and a CR rate of 88.9%. These long-term survival data, derived from a median follow-up of 77.6 months, provide critical insights into the durability of the IRM regimen, which is particularly notable given the historically poor prognosis of PCNS DLBCL. The extended follow-up period allowed us to capture late relapses and assess the sustainability of treatment responses—an essential consideration for a disease with a high risk of recurrence within the first two years. However, the interpretation of these results must be tempered by the study’s limitations. The cohort’s median age of 59 years (range: 48-66) and exclusion of patients over 70 years may have introduced selection bias, as younger patients generally tolerate intensive therapies better and exhibit improved survival outcomes.

The observed 5-year OS/PFS rates surpass those reported in many prior studies, suggesting potential advantages of combining BTK inhibition with HD-MTX-based chemotherapy. According to the NCCN Guidelines (Version 5.2024), the preferred induction regimens for PCNSL include high-dose methotrexate (8 g/m² or 3.5 g/m²) combined with rituximab-based protocols such as R-MPV (rituximab, methotrexate, procarbazine, and vincristine) or temozolomide (TMZ) with rituximab, often alongside consideration of WBRT (24). The MATRIX regimen in the IELSG32 trial achieved a 7-year OS of 70%, but was associated with significant toxicity, including severe bone marrow suppression and febrile neutropenia. Consequently, the MATRIX regimen was considered suitable for patients under the age of 60 years, in good physical condition, ensuring ample transfusion support, and benefiting from multidisciplinary consultation (25). Similarly, the PRECIS study reported an 8-year OS of 69% with whole-brain radiotherapy (WBRT) and 65% with high-dose chemotherapy plus ASCT, but these regimens also carried substantial toxicity risks (10). The HOVON 105/ALLG NHL prospective randomized phase III trial comparing MBVP and R-MBVP in initial PCNS DLBCL treatment reported CR rates of 36% for MBVP and 30% for R-MBVP. An update at the 2022 EHA meeting disclosed 82-month follow-up data with 5-year OS rates of 54% for MBVP and 70% for R-MBVP, along with 5-year PFS rates of 31% for MBVP and 57% for R-MBVP (9, 26). Recent studies incorporating BTK inhibitors have shown further improvements in efficacy. A study evaluating zanubrutinib, rituximab, lenalidomide, and TMZ in treatment-naïve PCNS DLBCL patients reported an ORR of 91.7% and a CR of 58.3%… (27) Similarly, another investigation that involved ibrutinib, HD-MTX, and TMZ regimens in 33 PCNS DLBCL patients documented an ORR of 94% and a complete response rate of 73% (28). A recently published study conducted a phase II trial of high - dose methotrexate plus ibrutinib and temozolomide in newly diagnosed PCNSL. A total of 35 patients were enrolled, with 33 included in the analysis. The best overall response rate was 93.9%, and the complete response rate was 72.7% for induction therapy. The 2 - year progression - free survival and overall survival were 57.6% and 84.8%, respectively. The incidence of grade≥3 adverse events was 27.3% (29). However, these trials had relatively short follow-up durations, limiting their ability to assess long-term outcomes such as OS and PFS.

The NCCN guidelines currently do not recommend maintenance therapy for PCNSL. However, emerging clinical evidence suggests that maintenance therapy may provide significant benefits, especially for elderly patients who are unable to undergo HDT-ASCT. A phase 2 study found ibrutinib maintenance (560 mg/day) was feasible and well-tolerated in elderly patients (60–85 years old) with newly-diagnosed PCNSL who achieved partial or complete response to first-line HD-MTX-based treatment. The 1- and 2-year PFS rates were 90% and 72.6%, respectively, and the 2-year OS rate was 89% (30). Another retrospective study showed lenalidomide maintenance significantly improved PFS and OS in patients with PR after induction (31). and time-limited strategies like HD-MTX or TMZ have shown promise in sustaining remission (32, 33). Our study also observed significant benefits from maintenance therapy in elderly patients not undergoing transplant. However, the optimal agents, dosages, and duration of maintenance therapy for PCNSL remain to be determined, and further research is needed to identify the patient subgroups that are most likely to benefit.

Molecular profiling in our study revealed mutations in NF-κB pathway-related genes (47% of cases), with *PIM1* (66.7%), *MYD88* (55.6%), *CD79B* (55.6%), and *GNA13* (22.2%) being the most frequently altered. While MYD88 and CD79B mutations are classically associated with BTK inhibitor sensitivity, their mutation rates in our cohort (55.6% for both) were lower than those reported in a meta-analysis (74% for *CD79B* and 50% for *MYD88*) (34). Considering that Ibrutinib monotherapy has demonstrated a response rate of up to 80% in the treatment of PCNS DLBCL (15), This discrepancy suggests that ibrutinib’s efficacy in PCNS DLBCL may extend beyond canonical BCR-NF-κB signaling. The tumor microenvironment (TME) likely contributes significantly to treatment response. as evidenced by the overexpression of immune checkpoint molecules like PD-L1 or PD-L2 on B-cell lymphomas, which evaded immune surveillance (35, 36). In multiple studies, ibrutinib downregulated the expression of negative immune checkpoint molecules and enhanced the T-cell function (37–39). Additionally, in other B-cell lymphomas, ibrutinib significantly increased the secretion of cytokines (IL-4, IL-6, IL-10, IL-17A, and TNF-α), sustainably improved the degranulation function of effector proteins, regulated the T cell differentiation and function, promoted T cell differentiation conducive to immune function restoration, and enhanced the T cell-mediated immune response, anti-tumor activity, and immune surveillance function (40, 41). These immunomodulatory effects may explain the high response rates observed in our cohort, particularly in tumors overexpressing PD-L1/PD-L2. However, the lack of TME profiling in this study limits direct mechanistic correlations.

This study has several limitations, including its small sample size (n=9), retrospective design, and lack of a control group. These factors may introduce selection bias and limit the generalizability of our findings. Additionally, the heterogeneity of molecular subtypes within our cohort, such as the case of a patient classified as MCD by NGS but GCB by Han’s classification, highlights the need for further investigation into the relationship between genetic subtypes and treatment response. Furthermore, due to the retrospective design of our study, standardized assessments of quality of life and cognitive function were not available, which limits our evaluation of the regimen’s impact on these important patient-centered outcomes. Although the IRM regimen appeared to be well-tolerated without severe myelosuppression-related complications such as major infections or hemorrhage—suggesting a potentially more favorable quality of life compared with conventional chemotherapy—this remains an observational impression rather than a systematically measured outcome. Prospective collection of these data will be incorporated into our future studies to provide a more comprehensive understanding of the treatment’s effects. Additionally, the heterogeneity in consolidation therapy (ASCT in 3 patients vs. additional cycles of IRM in 6 patients), while reflecting real-world clinical practice, may have introduced confounding factors in the long-term outcome analysis.

Beyond these limitations, novel tools may further enhance response assessment in PCNSL. Currently, imaging criteria (MRI and PET-CT) remain the standard, but interpretation can be challenging in cases with minimal residual enhancement or post-treatment changes. Liquid biopsy, particularly circulating tumor DNA (ctDNA), has recently emerged as a promising non-invasive tool for diagnosis, prognostication, and longitudinal monitoring in B- and T-cell lymphomas, including PCNSL, as comprehensively reviewed by Almasri et al. (42) Incorporating serial ctDNA analysis into future IRM trials could provide real-time insights into disease dynamics and may serve as an early surrogate marker of relapse.

Despite these limitations, our findings suggest that the IRM regimen is a promising first-line therapy for newly diagnosed PCNS DLBCL, particularly for patients ineligible for intensive chemotherapy or ASCT. The combination regimen of high response rates, favorable long-term outcomes, and manageable toxicity underscores its potential clinical utility. These preliminary findings warrant further investigation in a larger, prospective cohort to confirm the efficacy and safety of this regimen(#ChiCTR1900027811) Future research should also focus on personalized treatment strategies based on molecular subtypes to optimize outcomes for patients with PCNS DLBCL.

## Data Availability

The original contributions presented in the study are included in the article/supplementary material. Further inquiries can be directed to the corresponding author.
